# A deep learning-based algorithm for the detection of personal protective equipment

**DOI:** 10.1371/journal.pone.0322115

**Published:** 2025-05-29

**Authors:** Bo Tong, Guan Li, Xiangli Bu, Yang Wang, Xingchen Yu

**Affiliations:** 1 School of Electronic Information and Control, North China Institute of Science and Technology, Langfang City, China; 2 Key Laboratory of Brain-Computer Interface Technology Application of the Ministry of Emergency Management, Beijing, China; 3 Ministry of Emergency Management Big Data Center, Beijing, China; 4 School of Emergency Equipment, North China Institute of Science and Technology, Beijing, China; 5 Hebei Province Mine Equipment Safety Monitoring Key Laboratory, Langfang City, China; Macau University of Science and Technology, MACAO

## Abstract

Personal protective equipment (PPE) is critical for ensuring the safety of construction workers. However, site surveillance images from construction sites often feature multi-size and multi-scale targets, leading to low detection accuracy for PPE in existing models. To address this issue, this paper proposes an improved model based on YOLOv8n.By enriching feature diversity and enhancing the model’s adaptability to geometric transformations, the detection accuracy is improved.A Multi-Scale Group Convolution Module (MSGP) was designed to extract multi-level features using different convolution kernels. A Multi-Scale Feature Diffusion Pyramid Network (MFDPN) was developed, which aggregates multi-scale features through the Multiscale Feature Focus (MFF) module and diffuses them across scales, providing each scale with detailed contextual information. A customized Task Alignment Module was introduced to integrate interactive features, optimizing both classification and localization tasks. The DCNV2(Deformable Convolutional Networks v2) module was incorporated to handle geometric scale transformations by generating spatial offsets and feature masks from interactive features, thereby improving localization accuracy and dynamically selecting weights to enhance classification precision.The improved model incorporates rich multi-level and multi-scale features, allowing it to better adapt to tasks involving geometric transformations and aligning with the image data distribution in construction scenarios. Additionally, structured pruning techniques were applied to the model at varying levels, further reducing computational and parameter loads. Experimental results indicate that at a pruning level of 1.5, mAP@0.5 and mAP@0.5:0.95 improved by 3.9% and 4.6%, respectively, while computational load decreased by 21% and parameter count dropped by 53%. The proposed MFD-YOLO(1.5) model achieves significant progress in detecting personal protective equipment on construction sites, with a substantial reduction in parameter count, making it suitable for deployment on resource-constrained edge devices.

## Introduction

The construction industry has consistently been one of the most hazardous sectors due to its labor-intensive nature and dangerous working environments [1]. According to relevant statistics, over one-fifth of fatal workplace accidents in the European Union occurred in the construction industry in 2021 [2]. In China, there were 773 and 689 safety incidents in housing and municipal engineering in 2019 and 2020, resulting in 904 and 794 fatalities, respectively [3,4]. In the United States, the number of fatalities in the construction industry reached 1,008 in 2020 [5]. Approximately 30% of production accidents in the UK occur in the construction sector [6]. With the ongoing expansion of the construction industry in developing countries, the accident rate may continue to rise [7]. These data clearly indicate that it is imperative to focus on construction safety and take effective measures to ensure the safety of workers.

There are many potential hazards on construction sites, such as mechanical collisions, falls, electrocution, etc.[8]The majority of construction accidents are related to human factors, with over 80% of accidents stemming from unsafe worker behaviour [9–11]. The lack of necessary protective equipment is one of the main causes of serious casualties [12]. The correct use of personal protective equipment(PPE) can significantly safeguard the lives of workers. however, for a large construction project, it can be difficult to use manpower to carry out inspections [13,14]. With advances in computer vision (CV), AI has surpassed humans in some aspects when it comes to detecting unsafe behaviour [15]. At the same time, closed-circuit television (CCTV) at construction sites can capture a large amount of video and image data, providing objective conditions for computer vision-based personal equipment protection detection [8].

However, there are currently some problems in using CV for personal equipment protection for detection: (1) Detection accuracy is limited. Cameras in architectural scenes usually have a wide-angle field of view, resulting in captured images with large-scale variations of the target object, containing different levels of detail and feature information. Therefore, the algorithm model needs to have the capability to effectively handle features of varying sizes. In traditional deep learning, low-level features typically capture basic information and details, such as edges and textures, while high-level features are more abstract and complex representations derived from low-level features. As the network depth increases, some low-level information may be lost, leading to the loss of crucial details during the fusion of high- and low-level features, which consequently reduces detection accuracy. (2) The model is too large for deployment. Edge devices typically have limited resources, and constraints on memory and computational power necessitate that models be smaller in size and more efficient.

To enhance the accuracy of detection algorithms, Wang Zheng *et al*. [16]introduced cross-level path aggregation in the feature fusion structure, which reduced the loss of target feature information and effectively supported the detection of personal protective equipment in coal mines. However, there are significant differences between construction sites and coal mine scenarios. Coal mine environments tend to have darker backgrounds and limited scene sizes. In contrast, construction sites have complex backgrounds and larger areas, requiring wide-angle cameras to ensure a broad view of the target locations, necessitating the handling of images at various scales. Huang Guoyan *et al*. [17]employed a progressive adaptive feature fusion technique to effectively address the issue of information conflict in helmet detection, achieving improved accuracy while maintaining a fast inference speed.

In practical engineering applications, the processing of image data often requires real-time or near-real-time responses. Considering cost constraints, there is a need for a model with lower computational and memory requirements.

Lin Jiehua *et al*. [18]developed a lightweight decoupled detection head that reduces the model’s parameter count while enhancing its classification and localization capabilities. In a one-stage detector, the detection head typically accounts for about 50% of the total parameter count. Optimizing the detection head can significantly reduce the parameter size, making the model more suitable for deployment on edge embedded devices. Zhao Jingyi *et al*. [19]utilized channel pruning to significantly reduce the model’s parameter count and size, supporting efficient deployment. This paper presents the MFD-YOLO architecture to address the challenges of detecting personal protective equipment in construction scenarios. Its main contributions are as follows:

(1) A Bottleneck structure is designed that leverages the concepts of group convolution and pointwise convolution, sending feature maps into convolutions of varying sizes for feature extraction. The fused feature maps possess richer features, making them more suitable for the variable target information present in construction scene images.
(2) In the Neck section, a customized Multi-Scale Feature Aggregation Module and feature diffusion mechanism are implemented, ensuring that each scale of features possesses detailed contextual information. The feature aggregation module accepts input from three scales, utilizing depth convolutions of varying sizes to capture rich cross-scale information. It includes a non-parametric module and employs the diffusion mechanism to spread detailed contextual information to each detection scale.
(3) In the detection head section, Batch Normalization (BN) layers are replaced with better-performing Group Normalization (GN) layers, significantly reducing the parameter count through shared convolutions. A Scale layer is introduced to ensure consistency in target scale across the detection head. A customized task alignment structure enhances the interaction between classification and localization features. Additionally, the introduction of DCNV2 deformable convolutions adjusts spatial offsets and weights, improving localization performance. Dynamic feature selection based on interactive features further enhances classification performance.
(4) An adaptive network pruning method is employed for global pruning, assessing parameter importance and removing unimportant network parameters to reduce model size, decrease computational complexity, and enhance inference speed. After pruning, the model is fine-tuned to recover any potential performance loss.

The remainder of this paper is organized as follows: Sect 2 presents related research on PPE detection development. Sect 3 provides a detailed description of the proposed MFD-YOLO architecture. Sect 4 discusses the experiments related to model validation. Finally, Sect 5 summarizes the work presented in this paper.

## Related work

The use of personal protective equipment (PPE) is crucial for protecting workers, especially those working in hazardous areas, from injuries caused by sudden incidents [13]. Traditionally, safety inspectors verify workers’ PPE compliance using checklists to reduce workplace accidents [14]. However, this manual verification method is not only time-consuming and labor-intensive but also prone to oversight, and it cannot achieve real-time monitoring, leading to many potential safety hazards going undetected. With advancements in artificial intelligence technology and closed-circuit television on construction sites, more effective supervision methods have emerged.

### Detection of PPE wearing based on traditional sensors and machine learning

Sensor-based methods typically require workers to wear specific sensor devices and communication-enabled equipment, such as Global Positioning Systems (GPS), Ultra-Wideband (UWB), and Bluetooth [20–22]. The data collected by these sensors are processed and analyzed through Internet of Things (IoT) platforms [23]. For example, Riaz *et al*. monitored work environments in confined spaces using integrated Building Information Modeling (BiM) and wireless sensors to prevent workers from encountering time-sensitive situations [24]. Gómez-de-Gabriel *et al*. combined Bluetooth Low Energy (BLE) and Bayesian distance estimators to prompt and intervene in cases of improperly installed PPE or insufficient distance from tools [20]. Zhang *et al*. utilized Radio Frequency Identification (RFID) in conjunction with IoT to issue warnings when workers enter hazardous areas on construction sites, transmitting alert information to remote servers via LoRa technology [23]. However, sensor-based methods have limitations, such as reliance on the establishment of tags, which incurs additional costs for installation and maintenance [25], and the extra equipment may interfere with workers’ normal tasks[26].

Detection methods based on machine learning typically focus on identifying single devices within images. Rubaiyat *et al*. extracted differential features from images using Histogram of Oriented Gradients (HOG) and combined color information with Circular Hough Transform (CHT) to detect helmets [27]. Liu *et al*. employed a Support Vector Machine (SVM) model to classify images based on helmet usage [28]. Wu *et al*. constructed a hybrid operator based on color Local Binary Patterns (LBP), Hu Moment Invariants (HMI), and color histograms (CH) to extract features of helmets in various colors and classified four types of helmet usage using Hierarchical Support Vector Machines (H-SVM) [29]. These methods often focus on specific regions of the image, extracting information from critical areas and using the differences between these areas and their surroundings for classification. While effective for certain tasks, they generally suffer from low detection accuracy, slow inference speed, and poor model generalization, making them inadequate for the real-time and complex requirements of construction sites [16].

### Detection of PPE wearing based on object detection

Deep learning-based object detection algorithms are typically divided into two categories [30]: two-stage object detection algorithms and one-stage object detection algorithms. Two-stage algorithms, such as Region-based Convolutional Neural Networks (R-CNN) [31] and Faster R-CNN [32],offer high accuracy but have complex network structures, demanding significant computational resources and resulting in slower inference speeds, making them unsuitable for practical engineering applications [16]. In contrast, one-stage object detection algorithms, like YOLO (You Only Look Once) [33]and Single Shot MultiBox Detector (SSD) [34], are more suitable for real-world applications due to their higher speed and lower computational demands.

In practical applications, one-stage object detection algorithms perform particularly well. For instance, Nath *et al*. [35] significantly reduced model complexity while maintaining detection accuracy by replacing the backbone network of YOLOv4 with MobileNet v3. Wang Zhen [16] improved the feature fusion path, enhancing the accuracy of PPE detection in coal mine scenarios. Zhao *et al*. [19] applied network pruning techniques to reduce the parameter count of building recognition algorithm models, making them more suitable for deployment on edge devices and improving inference speed.

## Methods

### MFD-YOLO overview

**Fig 1:**
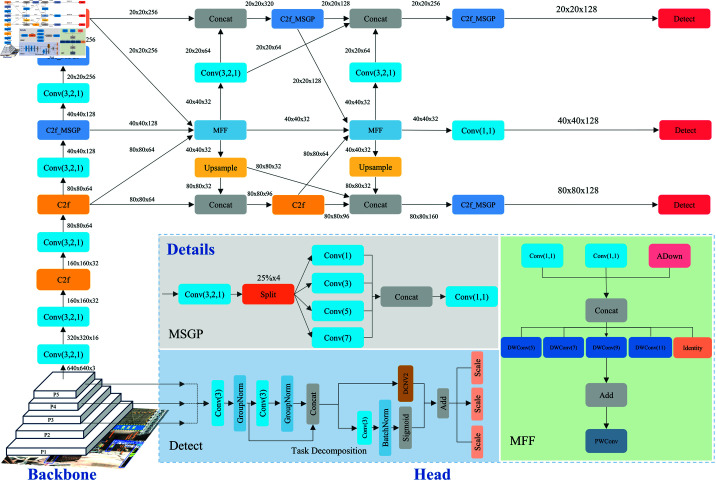
MFD-YOLO structure.

As a one-stage detector, YOLOv8 has demonstrated outstanding performance in the field of object detection, particularly showing great potential for personal protective equipment detection. Therefore, this paper proposes a PPE detection method based on YOLOv8n, referred to as MFD-YOLO, aiming to leverage the advantages of one-stage detectors while addressing the shortcomings of traditional methods. The model structure is illustrated in Fig 1.

In the MFD-YOLO architecture, we propose the MGPC (Multi-scale Group Pointwise Convolution) module, which utilizes convolutions of different sizes to extract features at various levels, enriching the feature flow. In the Neck section, we designed the MFF module to aggregate features from three scales, and through the Multiscale Feature Diffusion Pyramid Network (MFDPN), these aggregated features are diffused across the various detection scales, ensuring that each scale possesses detailed contextual information. In the detection head section, we designed the Dynamic Task-alignment Detection Heads (DTADH) module, implementing a customized task alignment mechanism. This mechanism aims to enhance the interaction between classification and localization tasks by generating spatial offsets and masks based on interactive features. To address the geometric deformation of detection targets, we introduced DCNv2 in the localization task. For the classification task, dynamic feature selection facilitates matching across multi-scale features. Finally, we performed global pruning on the model using adaptive pruning techniques, resulting in a model with a smaller parameter count and lower computational requirements.

### MGPC module

In construction scenarios, the diverse positioning of cameras often results in the acquisition of image data at varying scales. In the traditional YOLOv8 backbone network, the C2f module is utilized for feature extraction, where input features are first processed through a 1x1 convolution to reduce the channel count and lower computational load. The features are then sent into a Bottleneck structure, where a 3x3 convolution is employed for feature extraction, and residual connections are used to prevent gradient vanishing. However, this approach relies solely on the receptive field of the 3x3 convolution, resulting in insufficiently rich information extraction.

**Fig 2:**
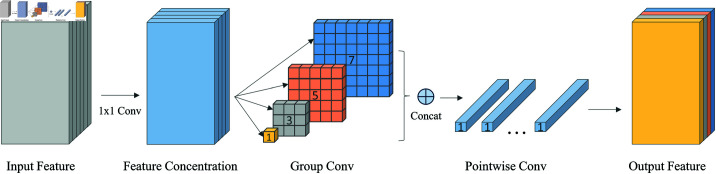
MGPC module.

To address this, we designed a new Bottleneck structure called Multi-scale Group Pointwise Convolution (MGPC), as illustrated in Fig 2. MGPC employs grouped convolution to divide the input feature channels into four groups, utilizing convolution kernels of sizes 1x1, 3x3, 5x5, and 7x7 to extract features and capture information at different levels, ultimately fusing the features through concatenation. Previous studies have shown that grouped convolution not only increases the diagonal correlation between filters but also reduces training parameters, making it less prone to overfitting [36]. The structure of MGPC is shown in Fig 2, with the parameters and computational load of traditional convolutions represented by the (Eq 1), assuming the input feature shape is [*C*_1_,*H*,*W]* and the output feature shape is $\left[C_{2},H^{\prime },W^{\prime }\right]$.

$$params=(k^{2}\times C_{1}+1)\times C_{2}$$ (1)

$$FLOPs=H^{\prime }\times W^{\prime }\times K^{2}\times C_{1}\times C_{2}$$ (2)

$$p_{up}(\upsilon_{i})\int_{‒\infty }^{s}\surd \frac{1}{\sqrt{2\pi }\sigma_{\upsilon_{i}}^{+}}e^{‒{\frac{(x‒\mu_{\upsilon_{i}}^{+}{)}^{2}}{2\sigma_{\upsilon_{i}}^{+}}}^{2}}dx$$ (3)

For grouped convolution, assuming it is divided into groups, the associated count is denoted as $(k^{2}\times \frac{C_{1}}{g}+1)\times \frac{C_{2}}{g}$ and the computational load in FLOPs is represented as $H^{\prime }\times W^{\prime }\times K^{2}\times \frac{C_{1}\times C_{2}}{g}$,Both parameters are reduced to varying extents depending on the size of the groups, which is advantageous for deployment on embedded devices. Additionally, the 1x1 convolution is a common method used to exchange channel information between grouped feature maps. By employing pointwise convolution, we enriched the inter-channel information. enhancing the expressiveness of the final feature map [37]. The MGPC structure combines features from different levels and channels,making it more suitable for the multi-scale transformation requirements in construction scenarios.

### MFDPN module

**Fig 3:**
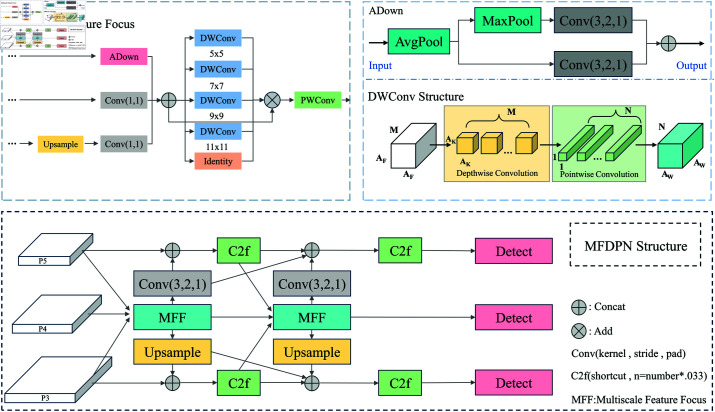
MFDPN module.

The image data obtained from CCTV presents challenges related to target scale variations and diverse contextual environments. PkiNet has been shown to effectively address scale transformations by extracting features within different receptive fields and collecting local contextual information [38]. Based on this concept, we designed the MFF module, which accepts input from three scales, P3, P4, and P5, as illustrated in Fig 3. When processing features from the P3 layer, we use an Adown module for downsampling, which retains more detailed information compared to traditional convolutions [39]. For the P5 layer, we first upsample to restore resolution and then apply a 1x1 convolution to adjust the channel count. The features from P3, P4, and P5 are then aggregated using Concat to form a fusion of multiscale features. The larger the receptive field, the richer the information obtained; thus, the aggregated features are passed through four different sizes of depth convolutions (5x5, 7x7, 9x9, and 11x11) alongwith a parameter-free Identity mapping. The Identity module, similar to a residual connection, maintains the continuity of the information flow. Subsequently, these features with varying receptive fields are fused with the previously aggregated features, and a 1×1 convolution is applied for further channel information integration. The resulting features are then re-fused with the aggregated features from the three scales, yielding features enriched with multilevel and detailed contextual information. Ultimately, the features containing multiscaleand detailed contextual information are fused again with the initial features from the three scales, enhancing the accuracy and robustness of the detection across each scale through upsampling, downsampling, and Concat operations.

### DTADH module

**Fig 4:**
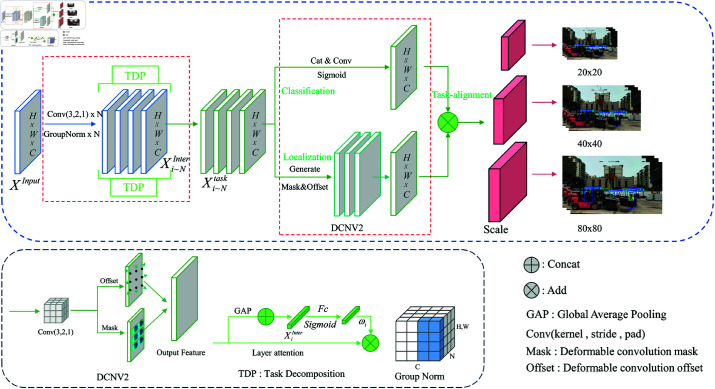
DTADH module.


In the design of the detection head for YOLOv8, classification and localization tasks operate independently. Due to their different learning mechanisms, there may be discrepancies in the spatial distribution of features, leading to biases during predictions [40]. To address this issue, we restructured the detection head by drawing inspiration from TOOD, as illustrated in Fig 4. Research indicates that Group Normalization (GN) can significantly enhance the performance of the detection head in both localization and classification tasks [41]. Consequently, we employed a 3x3 convolution combined with a GN layer as the feature extractor to derive interactive features from the P3, P4, and P5 layers. The input features processed by the Neck layer are represented X^*input*^,while the interactive features learned from the feature extractor are denoted as X^*Inter*^,where *H*, *W* and *C* correspond to the height, width, and number of channels, respectively. The Task Decomposition module utilizes a layer attention mechanism to compute the attention weights for each feature map ω_i_,Based on the varying levels of attention, features are mapped for subsequent independent processing in classification and localization tasks. The classification task dynamically adjusts the convolution weights based on the interactive features, generating classification predictions. The localization task uses the interactive features to produce spatial displacement offsets, denoted as *Offset*, along with masks *Masks*.By employing DCNv2 with the generated offsets and masks, the module effectively adjusts the spatial positions and weights of the feature maps, allowing it to better accommodate localization tasks with geometric transformations. DCNV2 introduces deformable convolutions that adaptively adjust receptive fields, allowing for better handling of objects with geometric variations and improving detection robustness. To reduce the model’s parameter count and facilitate deployment on resource-constrained edge devices, we implemented shared convolution techniques. This enables the feature extractor, task alignment processing, and subsequent classification and localization components to share parameters, as highlighted by the red dashed box in the diagram. Furthermore, we introduced Scale layers to ensure consistency in the target scales detected by each detection head, as detailed in Equations 5, 6, and 7.

$$\sigma (x)=\frac{1}{1+e^{‒x}}$$ (4)

$$\omega_{i}=\sigma (fc_{2}(\sigma (fc_{1}(X^{inter}))))$$ (5)

$$X_{i}^{task}=\omega_{i}.X_{i}^{inter},\forall i\in \{\begin{array}{c} 1,2,3...,N \end{array}\}$$ (6)

In the (Eq 4), $X_{i}^{task}$ denotes the task decomposition for each layer of features, while *fc*_1_ and *fc*_2_ represent the fully connected layers. σ is sigmoid. The interactive features $X_{i\sim N}^{Inter}$ undergo global average pooling to capture the contextual information of the feature maps, resulting in a global feature representation for each channel. These processed features are then concatenated to form ${\text{X}}^{\text{Inter}}$. The modified ${\text{X}}^{\text{Inter}}$ is fed into a fully connected layer to generate feature mappings. An activation function is applied to produce attention weights, which assess the importance of each layer’s features in the classification and localization tasks. Based on these attention weights, the interactive features are allocated to their respective tasks for further processing.

### Prune

In the context of embedded devices frequently facing resource limitations on construction sites, it is crucial to reduce both the model’s parameter count and computational load for effective deployment. Network pruning, a technique that reduces the size of neural networks by removing unimportant weights, can help minimize costs. This paper employs Layer-wise Adaptive Magnitude-based Pruning (LAMP) to perform structured pruning on the improved MFD-YOLO model [42]. LAMP pruning involves designing a scoring function to quantify the loss incurred on the output layer due to pruning. We select weights that have minimal impact on model performance for removal. During the pruning process, each layer’s weight *score* are calculated and ranked, with a global sparsity target set to remove the weights with the lowest *score* until the desired global pruning ratio *speedup* is achieved. Through this method, we effectively eliminate unimportant channels in the MFD-YOLO network, significantly reducing the model’s parameter count and computational complexity, as detailed in the following (Eq 7).

$$score\left(i;\omega \right)=\frac{{\left(\omega_{i}\right)}^{2}}{\sum_{n\geq i}{\left(\omega_{n}\right)}^{2}}$$ (7)

$$speedup=\frac{GFLOPs_{1}}{GFLOPs_{2}}$$ (8)

In the above equation, $\omega_{i}$ represents the weight of a particular layer, *i* denotes the index of that layer, n≥i signifies all weights in ascending order following the current weight $\omega_{i}$.The denominator in the formula indicates the sum of the squares of the remaining weights in the current layer, while the numerator represents the square of the current weight, assessing its overall importance to the model. LAMP prunes weights in ascending order of their *score* , with *speedup* denoting the pruning ratio. *GFLOPs*_1_ and *GFLOPs*_2_ represent the computational loads before and after pruning, respectively. this ratio is not strictly defined, and pruning typically stops when the ratio approaches a certain threshold.

$$\min_{M}\underset{\|x{\|}_{2}\leq 1}{\sup}\|f(x;W)-f(x;M⊙W){\|}_{2}$$ (9)

In LAMP pruning, the Frobenius norm is used to approximate the loss minimization problem. In the (Eq 9), f(x;ω) represents the output of a specific layer in the network model, where *x* is the input and *w* is the tensor of weights. *M* is a binary mask matrix indicating whether a layer participates in pruning. The product $r_{CD_{V}}\left(\vec{p},n\right)=rM\left(\vec{p},n\right)\;\omega_{cd}\left(\vec{p},n\right)$ yields the weight matrix after pruning. By quantifying the various impacts generated during the pruning process, adjustments are made to the weights of each layer to minimize the loss at the output layer, ensuring that the outputs remain as close as possible before and after pruning.

## Experiment and results

### Dataset and environment configuration

The experimental environment was configured as follows: Software—Operating System: Ubuntu 22.04; Python: 3.9; PyTorch: 2.1.2; Ultralytics: 8.2.50. Hardware—Processor: Intel(R) Xeon(R) Platinum 8352V at 2.1 GHz; RAM: 80 GB; GPU: Nvidia GeForce RTX 4090 with 24 GB. To enable a quantitative comparison of model performance before and after improvements, we standardized the training parameters, as detailed in Table 1.

**Table 1:** Parameter setting table.

Parameters	Parameters Settings
Epochs	300
Batch Size	16
Learning rate	0.0001
Weight decay	0.0005
Optimizer	SGD
Warmup	200
Pretrain	False

### Evaluation metrics

In this experiment, we evaluated the improved model based on detection accuracy, speed, and model size, using the following metrics: mean Average Precision (mAP), inference speed (FPS), number of parameters, and GFLOPs (Giga Floating Point Operations per second). The mAP is derived from the average precision (AP), which is related to precision (P) and recall (R). The (Eq 10) are as follows:

$$P=\frac{TP}{TP+FP}$$ (10)

$$R=\frac{TP}{TP+FP}$$ (11)

$$AP=\int_{0}^{1}PdR$$ (12)

$$mAP=\frac{1}{N}\sum_{i=1}^{N}AP_{i}$$ (13)

Where *TP*, *FP*, *FN* represent true positive samples, false positive samples, and false negative samples, respectively. The *AP* is the area under the curve formed by *P* and *R*, while the *mAP* is the average of the *AP* values across all classes, where *N* is the total number of detection classes. For a model to be deployable on edge devices, model size and inference speed are crucial evaluation metrics. We quantify model size using the number of parameters and assess inference speed using *FPS*. The formula is shown in (Eq 14),where *t*_1_, *t*_2_ and *t*_3_ represent the preprocessing time, inference time, and postprocessing time of the model on an image, respectively.

$$FPS=\frac{1000ms}{\left(t_{1}+t_{2}+t_{3}\right)}$$ (14)

### Ablation experiment

In this experiment, YOLOv8n was used as the baseline model. To validate the effectiveness of the proposed improvements, we conducted ablation experiments, comparing each modified module one by one and analyzing the results in detail. Our proposed model is named MFD-YOLO, and we provide the quantification results of certain modules along with a visual analysis in the results. The results of the ablation experiments are shown in Table 2, while the PR comparison chart of the model improvements is illustrated in Fig 6, where A represents the MGPC module, B represents the FDPN module, and C represents the DTADH module. The detection structures for each category are detailed in Table 3.

**Table 2:** Ablation experiment.

Model	F1	AUC-ROC	mAP@0.5	mAP@0.5:0.95	GFLOPs	Parameters	FPS
YOLOv8n	66.7%	72.8%	70.2%	35.1%	8.1	3006233	148.2
A	67.5%	73.3%	70.9%	35.4%	7.9	2871577	144.6
B	69.3%	74.7%	72.3%	38.6%	9.4	3042649	120.4
C	68.3%	73.9%	72.0%	36.2%	8.6	2240230	128.2
A+B	68.7%	74.1%	72.1%	38.4%	9.3	2952921	117.4
A+C	70.3%	77.8%	73.3%	38.9%	8.3	2128102	123.6
B+C	70.7%	78.3%	74.2%	40.0%	10.0	2606886	106.4
MFD-YOLO	70.3%	78.9%	74.4%	40.2%	9.7	2505958	102.5

**Table 3:** Detection results of each category.

Categories	Model	P(%)	R(%)	mAP@0.5(%)	mAP@0.5:0.95(%)
person	YOLOv8n	83.2	77.0	82.4	82.4
	MFD-YOLO	82.6	81.8	86.1	49.3
helmet	YOLOv8n	82.6	51.4	59.7	25.6
	MFD-YOLO	85.1	55.4	64.8	23.6
vest	YOLOv8n	83.5	64.2	68.6	37.1
	MFD-YOLO	85.4	66.6	72.3	41.7

MGPC Module. In Table 2, the mAP@0.5, mAP@0.5:0.95, and Parameters for the baseline model YOLOv8n before improvements were 70.2%, 35.1%, and 3,006,233, respectively. The GFLOPs and inference speed before the improvements were 8.1 and 158.7, respectively. After replacing part of the C2f Bottleneck in the network structure with MGPC, the parameter count and computation decreased by 0.2M and 0.2G, respectively, while the mAP@0.5 and mAP@0.5:0.95 increased by 0.7% and 0.3%. This indicates that using multi-scale features to enhance personal protective equipment detection in construction scenarios is effective. The changes in parameter count after introducing MGPC into the backbone are shown in Table 4. In networks of the same depth, using MGPC instead of C2f resulted in a reduction in both parameter count and computation, although the inference time slightly increased.

**Table 4:** Changes in improved parameters of the backbone layer.

Sequence	Modules	Parameters	GFLOPs	Time(ms)
6	Block.C2f	197632	0.64	18.57
	Block.MGPC	175360	0.57	21.31
8	Block.C2f	460288	0.37	9.35
	Block.MGPC	415488	0.33	9.81

MFDPN Module. By replacing the PAN+FPN structure in YOLOv8 with the multi-scale feature aggregation pyramid, the mAP@0.5 and mAP@0.5:0.95 increased by 2.1% and 3.5%, respectively. However, the increased complexity of the network structure also led to an increase in computation and parameter count, resulting in a decrease in model inference speed (FPS) by 27.8. The improvements to the Neck enhance the richness of feature information, as illustrated in the heatmap shown in Fig 7, demonstrating that MFDPN improves detection performance across different scales.

**Fig 5:**
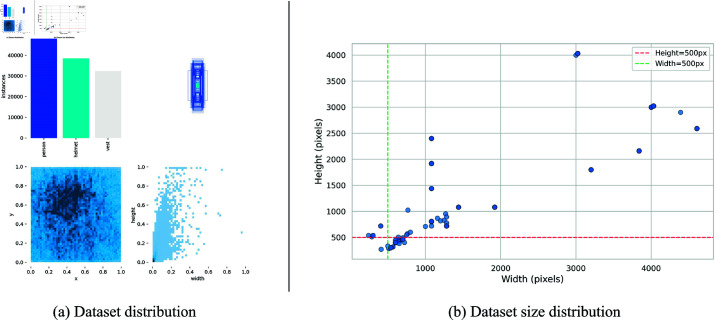
Dataset distribution. (a) Dataset distribution, (b) dataset size distribution.

**Fig 6:**
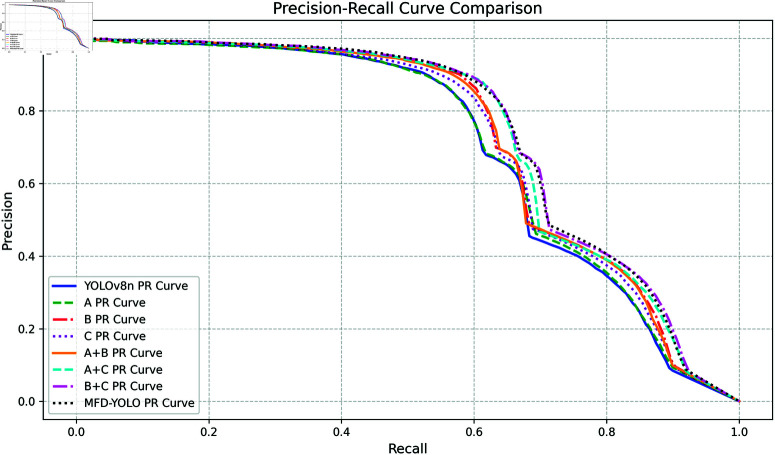
PR comparison chart. (a) Dataset distribution, (b) dataset size distribution.

**Fig 7:**
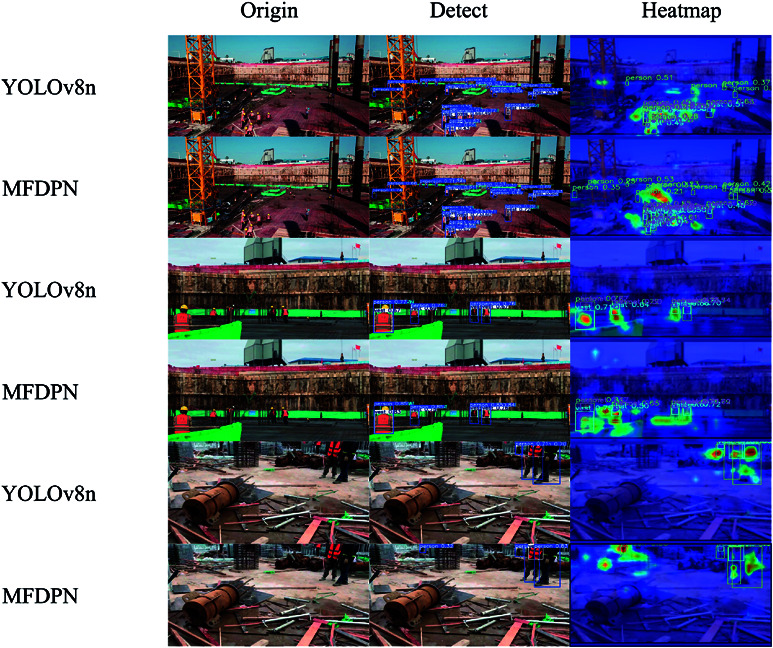
Comparison of detection effects. Origin is the original image,Detect is the detection result,Heatmap is the detection heatmap.

DTADH Module. The interaction features obtained through feature fusion allow the detection head’s localization task to generate relevant spatial offsets and masks based on these interaction features, while the classification task dynamically selects weights based on the same features. The introduction of DCNv2 and Scale layers better accommodates geometric transformations in images. We separated the test images by size and predicted the images corresponding to the top four size proportions. The detection results, as shown in Table 6, indicate that the DTADH module improves detection accuracy across different sizes compared to the original model.

**Table 5:** Detection effect of different sizes.

Dimensions	Model	P(%)	R(%)	mAP@0.5(%)	mAP@0.5:0.95(%)
1920x1080	YOLOv8n	84.2	74.6	80.2	80.2
	DTADH	84.8	75.9	81.8	41.9
1440x1080	YOLOv8n	90.3	79.7	87.6	51.2
	DTADH	90.5	82.6	88.2	52.2
3840x2160	YOLOv8n	74.0	35.0	41.6	17.0
	DTADH	75.7	36.4	43.7	18.8
4032x3024	YOLOv8n	93.1	65.7	75.7	45.7
	DTADH	93.6	69.5	76.5	46.5

**Table 6:** Multi scale detection.

Scale	Model	P(%)	R(%)	mAP@0.5(%)	mAP@0.5:0.95(%)
small	YOLOv8n	78.7	51.4	61.4	25.4
	MFD-YOLO	81.3	58.8	64.1	27.3
medium	YOLOv8n	79.6	74.3	59.7	31.6
	MFD-YOLO	82.3	78.5	66.5	35.7
large	YOLOv8n	83.5	64.2	72.6	37.1
	MFD-YOLO	87.4	68.6	76.3	42.5

After introducing the DTADH module, mAP@0.5 and mAP@0.5:0.95 increased by 1.8% and 1.1%, respectively. Although the computational load slightly increased due to a uniform number of channels, the parameter count decreased by 25%, and the inference speed (FPS) dropped by 20. Overall, the improved model showed enhancements of 4.2% and 5.1% in mAP0.5 and mAP0.5:0.95, respectively, while reducing the parameter count by 17%. This modified network not only lowered the parameter count but also increased the detection accuracy of personal protective equipment for workers. The GFLOPs increased by 1.6 due to the higher complexity of the network; the designed modules required more computation than the detection part of YOLOv8n, resulting in a final FPS decrease of 45.7%.

To further validate the multi-scale detection capability of MFD-YOLO, we conducted additional experiments on the CHV dataset, following the COCO evaluation criteria to classify objects into three categories based on their bounding box area: small ($\text{area}<1024\;{\text{px}}^{2}$), medium ($1024\text{pxv}\leq \text{area}<9216\;{\text{px}}^{2}$), and large ($\text{area}\geq 9216\;{\text{px}}^{2}$). We then computed small, medium, and large to quantitatively assess the model’s performance across different object scales. The results, presented in Table 6 , demonstrate that MFD-YOLO consistently outperforms the baseline model across all object sizes, further substantiating its effectiveness in multi-scale object detection. This additional analysis strengthens the claim regarding our model’s capability to handle objects of varying sizes.

### Pruning experiment

**Fig 8:**
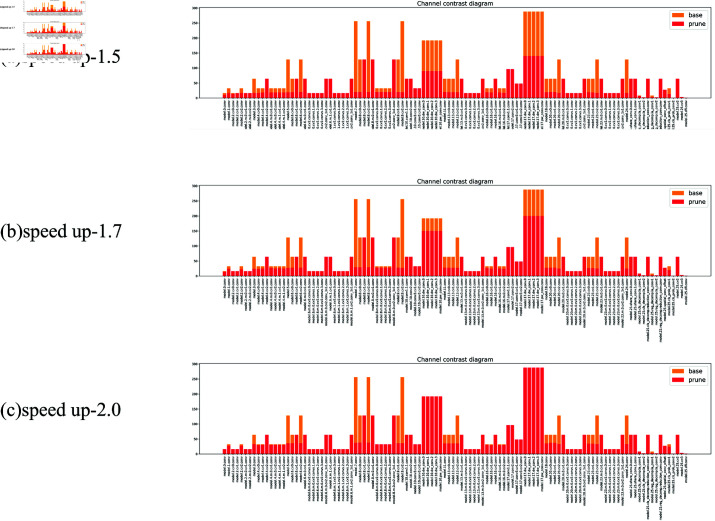
Proportion of pruning. (a) Speed up 1.5, (b) speed up 1.7, (c) speed up 2.0.

Although the improved model enhances target detection accuracy, it also leads to an increase in computational load. Reducing this computational burden and improving inference speed are especially critical for resource-constrained embedded devices. We employed an adaptive pruning approach to remove unimportant channels, thereby reducing both the parameter count and computational complexity. The results of the pruning experiments are shown in Table 7, and the channel comparisons of each module before and after pruning are illustrated in Fig 8.

**Table 7:** Pruning experiment.

Model	mAP@0.5	mAP@0.5:0.95	GFLOPs	Parameters	FPS
MFD-YOLO	74.4%	40.2%	9.7	2505958	102.5
SpeedUp-1.5	74.1%	39.7%	6.4	1365464	131.3
SpeedUp-1.7	71.6%	37.4%	5.7	1231063	135.3
SpeedUp-2.0	68.6%	33.1%	4.8	1085933	129.6

After applying the LAMP method for structured pruning, the channel counts of various modules were reduced, with some modules completely pruned. At a pruning ratio of 1.5, the model’s performance slightly decreased by 0.3% and 0.5% for mAP@0.5 and mAP@0.5:0.95, respectively, while the computational load decreased by 34%, and the parameter count dropped by 45%, leading to a 28.8% increase in FPS.

At a pruning ratio of 1.7, performance metrics for mAP@0.5 and mAP@0.5:0.95 dropped by 2.8% for both, with a 42% reduction in computational load and a 50% decrease in parameters, resulting in a 32.8% increase in FPS. At a pruning ratio of 2, the performance further declined by 5.8% and 7.1% for mAP@0.5 and mAP@0.5:0.95, respectively, with a 50% drop in computational load, a 56% decrease in parameters, and a 27.1% increase in FPS. Overall, when comparing mAP@0.5 alongside computational load, parameter count, and FPS, the pruning ratio of 1.5 yielded the best results.

### Comparative experiment

**Fig 9:**
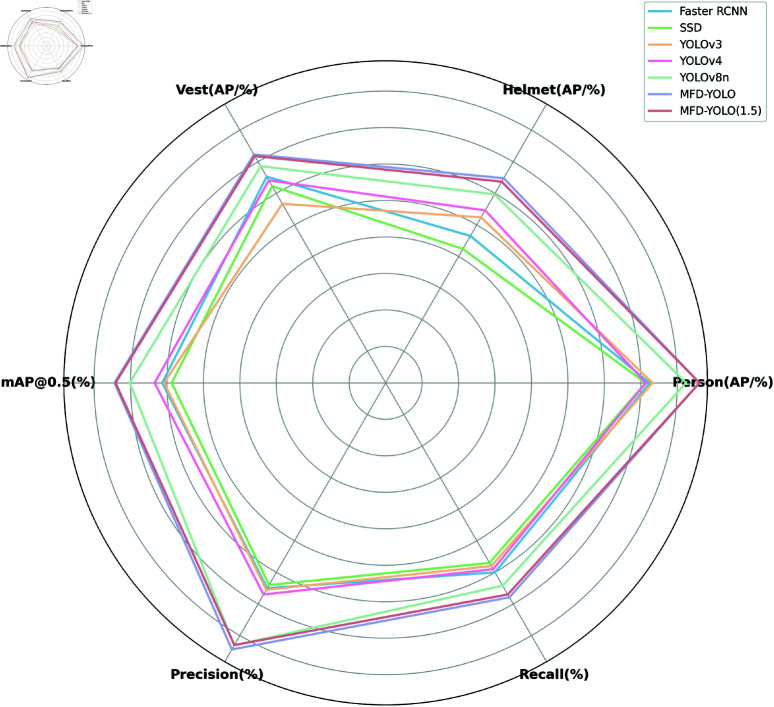
Radar chart comparing indicators.

To better illustrate the advantages of the improved MFD-YOLOv8 detection model, we conducted comparative experiments. We selected currently advanced detection algorithms as comparison models [44]. The model size was quantified using parameter counts, while GFLOPs and FPS were used to estimate inference speed. For evaluation of detection accuracy, we chose AP and mAP@0.5(%) as the metrics. The results of the comparative experiments are shown in Table 8, with a radar chart comparing other metrics depicted in Fig 9.

**Table 8:** Comparative experiment.

Model	mAP@0.5(%)	GFLOPs	Parameters(M)	FPS
Faster RCNN	61.3%	257.3	62.4	21.3
SSD	58.7%	52.7	37.8	37.8
YOLOv3	60.8%	155.3	62.1	43.4
YOLOv4	63.4%	140.0	64.6	32.3
YOLOv5n	68.8%	7.1	2.6	147.3
YOLOv8n	70.2%	8.1	3.0	148.2
Mobile-YOLOv8n	64.8%	7.8	5.8	139.5
YOLOv11n	70.0 %	6.5	2.6	129.7
MFD-YOLO	74.4%	9.7	2.5	102.5
MFD-YOLO(1.5)	74.1%	6.4	1.4	131.3

The mAP values for Faster RCNN and SSD algorithms are 61.3% and 58.7%, respectively, with GFLOPs values of 257.3 and 52.7, and FPS values of 21.3 and 47.2. These algorithms exhibit low detection accuracy for personal protective equipment and have high computational complexity. YOLOv3 has an mAP of 60.8%, GFLOPs of 155.3, and a parameter count of 62.1M, resulting in a detection speed of 43.4. Its relatively high parameter count leads to significant memory usage. YOLOv4 achieves an mAP of 63.4%, surpassing earlier algorithms, but still has considerable computational and parameter demands, reaching 140 GFLOPs and 64.6M parameters. YOLOv5n is a commonly used model with a detection accuracy of 68.8%, a parameter count of 2.6M, and a computational complexity of 7.1 GFLOPs. However, its accuracy is lower compared to YOLOv8, and due to its reliance on anchor boxes and a coupled detection head, it presents fewer advantages for further improvements. We also experimented with replacing YOLOv8n’s backbone with the widely used lightweight MobileNet architecture. This resulted in an mAP of 64.8%, with a parameter count of 5.8M and a computational complexity of 7.8 GFLOPs. Although this modification reduced both the parameter count and computational cost compared to YOLOv8n, it led to a significant drop in accuracy. Additionally, we evaluated the latest YOLOv11n model, which achieved an average detection accuracy of 70.0%, with a parameter count of 2.6M and a computational complexity of 6.5 GFLOPs. Despite its lower parameter count and computational cost, its inference speed (FPS) was lower than that of YOLOv8n, making it less suitable for real-world deployment.YOLOv8n achieves an mAP of 70.2%, with 2.5M parameters, 9.7 GFLOPs, and an FPS of 148.2. Although it performs well, its computational load and parameter count remain somewhat high for deployment on edge embedded devices. The improved model, MFD-YOLO, reaches a maximum mAP of 74.4, with a computational load of 9.7 GFLOPs, 2.5M parameters, and an FPS of 102.5. Compared to YOLOv8n, while the parameter count has decreased, the computational load has increased, resulting in slower detection speeds, which is also not ideal for deployment. After applying a pruning ratio of 1.5, we obtained MFD-YOLO(1.5). Compared to YOLOv8n, the mAP increased by 3.9%, while the computational load decreased by 21% and the parameter count reduced by 53%. Although the FPS decreased by 16.9, this has a minimal impact on real-time detection tasks. The radar chart demonstrates that MFD-YOLO achieves optimal detection across various categories. Additionally, the pruned MFD-YOLO addresses the issue of high computational load, meeting the requirements for real-time detection of personal protective equipment in construction scenarios, while occupying less memory and maintaining a high running speed. Overall, the algorithm obtained after pruning is superior to other mainstream algorithms.

### Algorithm deployment

To evaluate the real-world performance of our proposed model, we deployed it on a Jetson Nano B1 edge computing device. Both the YOLOv8n and MFD-YOLOv8n(1.5) models were deployed and tested under two lighting conditions: well-lit and low-light environments.

**Fig 10:**
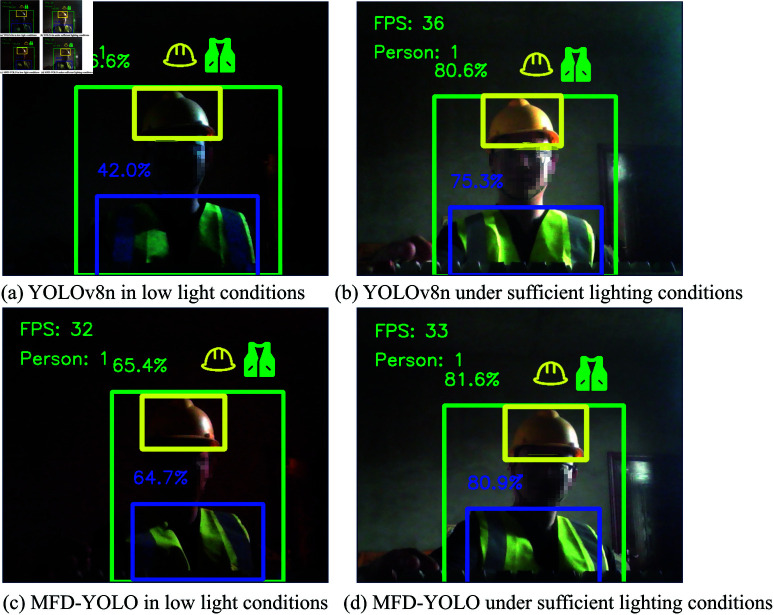
Algorithm deployment. (a) YOLOv8n in low light conditions, (b) YOLOv8n under sufficient lighting conditions, (c) MFD-YOLO in low light conditions, (d) MFD-YOLO under sufficient lighting conditions.

For performance evaluation, we used FPS (frames per second) as the metric for inference speed and AP (average precision) as the metric for detection accuracy. The FPS and AP values were averaged over 100 consecutive frames to obtain a comprehensive assessment. The summarized results are presented in the table below, while sample detection images from the real-world deployment are shown in the accompanying Fig 10.

(a) shows the detection results of YOLOv8n under low-light conditions, while (b) presents the detection results of YOLOv8n under well-lit conditions. It can be observed that the detection accuracy decreases under low-light conditions. (c) and (d) display the detection results of MFD-YOLO (1.5) under low-light and well-lit conditions, respectively. Compared to YOLOv8n, the detection accuracy of MFD-YOLO (1.5) improves in both scenarios. The average FPS for YOLOv8n is 35, whereas for MFD-YOLO (1.5), it is 30. For edge detection tasks, this difference is entirely acceptable.

## Discussion and conclusion

In this study, we proposed an improved model for detecting personal protective equipment (PPE) worn on construction sites, based on YOLOv8n. The goal is to address issues such as low detection accuracy and difficulties in model deployment. The main improvements include:

(1)Designed the MSGP module, which utilizes grouped convolutions of varying sizes to extract multi-level features while reducing the number of parameters and computational load, thereby enhancing feature representation.

(2)Constructed the MFDPN (Multi-scale Feature Diffusion Pyramid Network) structure, which aggregates feature information from the P3, P4, and P5 layers through the MFF module. A feature fusion pathway spreads detailed contextual information across various detection layers, improving the model’s capability to handle targets of different scales.

(3)Customized a task alignment mechanism that separates classification and localization tasks. Introduced DCNv2 to adjust spatial positions and weights, allowing the localization task to better adapt to the geometric transformations of targets at different scales. The classification task dynamically adjusts weights based on interaction features and utilizes the Scale layer to ensure consistency in detection results.

(4)Employed an adaptive structured pruning method to trim the improved model, removing redundant network parameters and reducing both the parameter count and computational load.

Ablation experiments and comparative tests demonstrated the effectiveness of the proposed improvements. The performance of each module was quantified and visualized during the experiments. Ultimately, MFD-YOLO(1.5) successfully reduced both the parameter count and computational load while effectively enhancing the accuracy of personal protective equipment detection on construction sites, thereby ensuring the safety of workers during construction activities.

Despite the improvements in detection accuracy across various scales, there are still instances of missed detections in specific scenarios, such as target occlusion and personnel overlap. Future work will focus on designing new attention mechanisms to enhance the model’s ability to recognize challenging samples. Additionally, considering the difficulties in data collection for construction scenarios, future efforts could incorporate GANs and anomaly generation modules to create synthetic detection datasets, effectively addressing the issue of insufficient data for specific tasks.
